# Correlation of human sperm centrosomal proteins with fertility

**DOI:** 10.4103/0974-1208.69344

**Published:** 2010

**Authors:** Indira Hinduja, Nishitha B Baliga, Kusum Zaveri

**Affiliations:** 1 Jaslok Hospital and Research Centre, Mehta Bhavan, Mumbai, India; 2 Inkus IVF Centre, Mehta Bhavan, Mumbai, India

**Keywords:** Centrosome, centrin, human sperm, protein, tubulin

## Abstract

**OBJECTIVE:**

The centrosome is the microtubule organizing center (MTOC) paternally inherited by the zygote during fertilization. As the centrosome is located in the midpiece of the sperm tail, we presume that oligoasthenozoospermic sperm samples should also have abnormal concentrations of centrosomal proteins. This study therefore aims to determine if there is any correlation between sperm centrosomal proteins, centrin, α and γ-tubulin, in sperm samples from normozoospermic and oligoasthenozoospermic men.

**MATERIALS AND METHODS:**

Proteins were extracted from the normozoospermic and oligoasthenozoospermic sperm samples and analyzed by Western Blot and ELISA for centrin, α and γ-tubulin.

**RESULTS:**

The levels of centrin, α and γ-tubulin are markedly lower in oligoasthenozoospermic sperm samples as compared to the normozoospermic sperm samples.

**CONCLUSIONS:**

Lower centrosomal protein expression in sperm samples of oligoasthenozoospermic infertile males may be a possible cause for their reduced fertility status. Further studies on these proteins are warranted to design rational approaches for the diagnosis and treatment of male infertility.

## INTRODUCTION

The centrosome is a structural component of mammalian cells which acts as scaffold on which a number of regulatory proteins are anchored. The centrosome is composed of a pair of centrioles surrounded by radiating strands of microtubules called the asters and plays a key role in cell division.

As early as 1887, it was postulated that a mature oocyte contains all the elements necessary for embryonic development with the exception of centrosome while the sperm contains the centrosome but lacks the substrate in which to operate. The sperm centrosome consists of two centrioles located in the midpiece of the sperm tail in a perpendicular arrangement and pericentriolar material. The centrosome is considered to be responsible for nucleation of microtubules and the formation of the mitotic spindle.

There is a paternal pattern of inheritance of the centrosome in humans. Human oocytes lack centrioles but the spermatozoa carry two. During ICSI when the sperm is injected into the oocyte, the sperm tail gets incorporated into the ooplasm. The centriolar region of the sperm forms the sperm aster while the sperm head decondenses; it is this aster that guides the female pronucleus toward the male pronucleus. The centriole duplicates during the pronuclear stage, and at syngamy they are found at opposite poles of the first cleavage.[1] The integrity of the centrosome is therefore crucial for successful fertilization and embryonic development. It is on this sperm centrosomal material that the oocyte centrosomal proteins assemble after sperm incorporation to form the sperm aster that is essential for uniting sperm and oocyte pronuclei. Therefore, any dysfunction of the sperm centrosome, or any alteration or perturbation associated with the centrosome, would have a detrimental impact on fertilization and embryonic development. Previous studies have demonstrated that sperm centriole controls the first mitotic divisions after fertilization.[2] Therefore, abnormalities in sperm centrosome would impair fertilization and embryonic development. As the centrosome is located in the sperm tail, it is likely that any abnormality in centrosome would result in impaired sperm motility. Thus, it becomes pertinent to study the centrosomal proteins in human spermatozoa in normozoospermic as well as oligoasthenozoospermic men.

Over 500 proteins have been associated with the centrosome. Studies with other cell systems have shown that tubulin, centrin – a calcium binding protein, γ globulin, in addition to the other proteins, are implicated in microtubule function and could play an important role in centrosome function. The aim of the present study has been to investigate differential expression of the sperm centrosomal proteins namely tubulin and centrin in the sperm samples of normospermic and oligoasthenospermic (OA) men.

## MATERIALS AND METHODS

### Collection of semen samples

Semen samples were collected by masturbation after three days of sexual abstinence from healthy proven fertile (donors who had fathered a child within the last two years) normozoospermic males (Group 1, *n*=20), and from oligoasthenozoospermic (OA) males (Group 2, *n*=20). The sperm concentration was >20×10^6^/ml with >50% grade a+b motility (rapid progressive and slow progressive motility) in Group 1 while the sperm concentration was <5×10^6^/ml with < 50% grade c+d (non progressive and immotile sperm) motility in Group 2. Each of the males from Group 2 had undergone semen analysis at least two to three times before enrolling them for the study.

Semen analysis was done according to the World Health Organization guidelines.[3] Written informed consent was obtained from all patients and donors. Institutional review board’s approval was obtained before undertaking the study.

### Processing of semen samples

The semen samples were washed three times with phosphate buffered saline (PBS), sperm concentration was adjusted to 1×10^6^ with 0.01M PBS (pH 7.5).[4] The motility and viability of the sperms in the washed and diluted samples were determined. Motile sperm separated by using swim up method from both normozoospermic and oligoasthenozoospermic males were used to compare centrin, α tubulin and γ–tubulin expressions in our study.

### Sperm treatment

Centrosome components are present in low quantities and often go undetected in morphologic and biochemical assays. The present study used centrosome isolating protocols to isolate centrosome components.[5–7] Briefly, 1×10^6^ motile spermatozoa were suspended in 0.01M PBS (pH7.5). The microtubules and microfilaments were depolymerized to release centrosome from the nucleus and cytoplasm by incubating the sperm suspension with 1 µg/ml cytochalasin D and 0.2 µM nocodazole for 1 h at 37°C in 5% CO_2_ incubator. The microtubules and actin filaments were rapidly and effectively depolymerized in most cells without affecting the centrosome structure, confirmed with immnunofluorescence microscopy.

### Sperm washing and lysis

The previous sperm suspension was centrifuged at 1200 g for 10 min and then washed with 0.01M Tris buffered saline (TBS) followed by a second wash with TBS in 0.1% sucrose buffer. A drop of this suspension was observed under the microscope to confirm that no sperm tails were isolated along with the centrosomal fractions.

The spermatozoa were then lysed in a solution of 1mM hydroxymethyl piperazine- 2 ethane sulfonic acid, (HEPES, pH 7.2), 0.5% NP-40, 0.5mM MgCl_2_, 0.1% β mercaptoethanol with protease inhibitors. Swollen nuclei and chromatin aggregates were removed by centrifugation at 2500 g for 10 min, and the supernatant was filtered through 50 µm nylon mesh (Small Parts Inc., U.S.A.). The supernatant containing lysed spermatozoa was then incubated for 30 min on ice in fresh HEPES solution adjusted to 10M and DNase 1 (2U/ ml, Sigma Chemicals, U.S.A).

### Isolation of centrosomal proteins

The lysate was incubated with 60% sucrose solution (w/v) containing 10 mM piperazine - N – N - bis ethane sulfonic acid (PIPES, pH 7.2), 0.1% Triton X-100, 0.1% β mercaptoethanol. The centrosomes were sedimented onto the sucrose cushion by centrifugation at 10,000 g in Sorvall cold centrifuge for 30 min. After the centrosomes were concentrated onto the 60% sucrose cushion, the supernatant was removed until only 250 µl-350 µl remained at the bottom of the tube. This crude preparation was further purified by discontinuous sucrose gradient centrifugation[5–7] consisting of 500 µl of 70%, 300 µl of 50% and 300 µl of 40% sucrose solution. The crude preparation was centrifuged for 1 h at 1,20,000g. Fractions (170 µl/fraction) were collected from the bottom. Each fraction was diluted in 1 ml of 10 mM PIPES buffer (700 µl of 70%, 500 µl of 50% and 500 µl of 40% sucrose solution).

### Qualitative protocol[5–7]

#### Western blotting

Two-twelve fractions were collected of which sucrose fractions 2-9 contained most of centrosomal proteins were used for SDS-PAGE and Western blot analysis. The remaining fractions did not show detectable quantities of these proteins. Fractions enriched for centrosomes indicated the presence of tubulin. The proteins were quantitated by Folin Lowry method. 80 microgram of protein was loaded in each well. The gel was stained by classical silver staining method.

All fractions were checked for α, γ–tubulin and centrin proteins. α and γ-tubulin analysis was done according to the method of Towbin *et al*.[8] Centrin was analyzed according to the method of Hulen *et al*.[9]

For Centrin Western Blotting, SDS poly acrylamide gel was soaked in KP buffer (25 mM KH_2_PO_4_/K_2_HPO_4_ buffer) pH 7.0. The PVDF (Polyvinylidene fluoride) membrane was pre-wetted briefly with absolute methanol and then rinsed for 15 mins in KP buffer. Transfer was conducted in KP buffer at 20 V overnight at 4°C (Hoeffer TE Transphor unit). After transfer, the membrane was incubated with 0.2% glutaraldehyde freshly prepared in KP buffer for 1 h. Glutaraldehyde fixation can detect as little as 0.1 ng of centrin. Membrane was then rinsed in KP buffer and then blocked in 5% Non Fat Dry Milk (NFDM) in TBS at room temperature for 2 h. Membrane was then incubated with TBS containing 0.5% Tween-20 (3 rinses) for 10 mins each at room temperature. Later on, the membrane was incubated with primary antibody (anti-centrin) diluted 1:200 in TBS overnight at 4°C. Rinsed with TBS-0.5% Tween-20 as above. Lastly, blots were incubated with anti mouse IgG, peroxidase conjugated goat secondary antibody (Banglore genei) diluted 1-500 in TBS for an hour at room temperature. Membrane was rinsed as above. The detection was done using diamino benzidine (DAB) hydrogen peroxide solution as chromogen and reaction was stopped with D/W. For negative control, monoclonal antibody was substituted with 0.01M PBS.

For α and γ-tubulin analysis, after SDS-PAGE, the gels were placed in Towbin’s transfer buffer for 5 min. Proteins were transferred on a nitrocellulose membrane (Amersham Life Sciences) at 20V, 4°C for 15-18 hours in a tank of transfer buffer. The membranes were blocked with 5% NFDM in PBS for 2 hours at room temperature. The membrane was then incubated in primary antibody (1:200 mouse monoclonal anti-α tubulin, anti-γ-tubulin 1:100) overnight at 4°C. Further washing was done with PBS containing 0.05% Tween-20 (three rinses) 10 mins each at room temperature. Lastly, the membrane was incubated with HRP linked secondary antibody at room temperature for 1 hour. The proteins were visualized as above. For negative control, monoclonal antibody was substituted with the 0.01 PBS. Commercially available purified α tubulin which is a 55-kDa protein from bovine brain (Sigma chemicals, USA) was used as positive control for α tubulin.

### Quantitation protocol

#### Enzyme linked immunosorbent assay

Cell enzyme linked immunosorbent assay (ELISA) was used to quantify the centrosomal centrin, α tubulin and γ-tubulin in normozoospermic and OA males.

The enzyme linked immunosorbent assay (ELISA) for centrosomal proteins was done by modifying the method reported by Malaviya *et al*.[10] In brief, the semen sample was washed thrice in PBS. Sperm cells at concentration of 1×10^6^ were adjusted with 0.01M PBS (pH 7.5). The plates were incubated at 37° C overnight. To each well was added 0.25% glutaraldehyde to fix the cells and incubated for 1 hour at 37°C. Three washings with 0.1% Tween PBS was given for 5 mins each. The cells were then blocked with 1% BSA-PBS for 1 hour at 37°C. The cells were then incubated with respective primary antibodies (anti-α tubulin DM1A, 1:200, Anti-γ-tubulin GTU88, 1:100, anti centrin 20H5, 1:100) prepared in 0.01M PBS, overnight at 4°C. Three washes with 0.1% Tween PBS of 5 mins each was given. Further, the cells were incubated with secondary antibody conjugated with horseradish peroxidase goat anti- mouse IgG (1:200) for 1 hour at 37°C, followed by three washes with 0.1% Tween PBS of 5 mins each.

Bound peroxidase was visualized by adding 200 µl ortho phenyl diamine (OPD) substrate solution (8 mg OPD) +0.03% H_2_O_2_ in 0.1 M citric acid and 0.2 M sodium hydrogen orthophosphate) and incubated in the dark for 20 mins.

The reaction was terminated by adding 100 µl of 4 N H_2_SO_4_ and calorimetric readings were taken at 490 nm in a titertek multiscan plate reader (Titertek). Monoclonal antibody was substituted with PBS - (0.01 M) for negative controls.

### Statistical analysis

Statistical analysis between group 1 and group 2 i.e. normozoospermic and oligoasthenozoospermic was done using Student’s *t*–test and *P*<0.05 was considered to be significant.

## RESULTS

### Silver staining analysis of human centrosomal proteins

**Figure 1:**
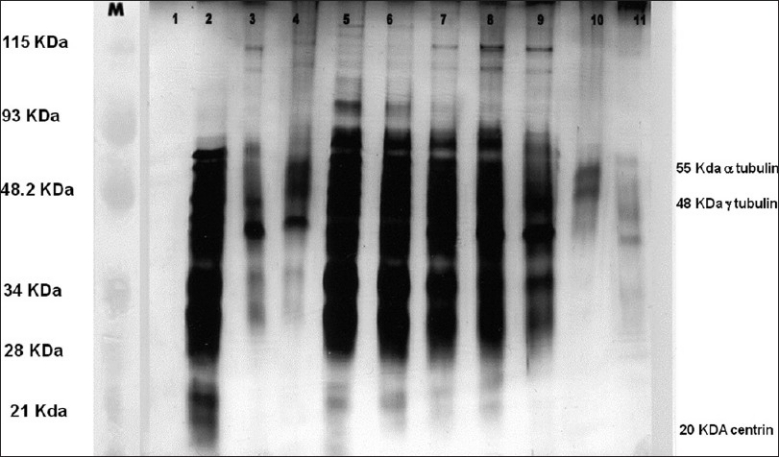
Silver staining of isolated sperm centrosomal fractions on a 12% SDS-PAGE. Lanes 1 to 11 - The various fractions collected by discontinuous sucrose density gradient method. Fractions 2-9 contains maximum proteins i.e., centrin, α and γ tubulin. Lane M - Broad range rainbow protein molecular weight marker (12,000 - 2,25,000, Amersham Biosciences now GE Healthcare Lifesciences)

The protein profile as seen on gel electrophoresis of the sucrose gradient fraction is shown in Figure 1. Fractions 2-9 contained most of the centrosomal proteins of our interest. The fractions demonstrated two major groups of protein, one of high molecular weight in the range of 45-65 kDa which included the centriolar tubulin and the other a very low molecular weight (20 kDa) protein, centrin. The proteins were further analyzed by Western blot analysis and ELISA in samples from normozoospermic and OA males.

### Western blot analysis

**Figure 2:**
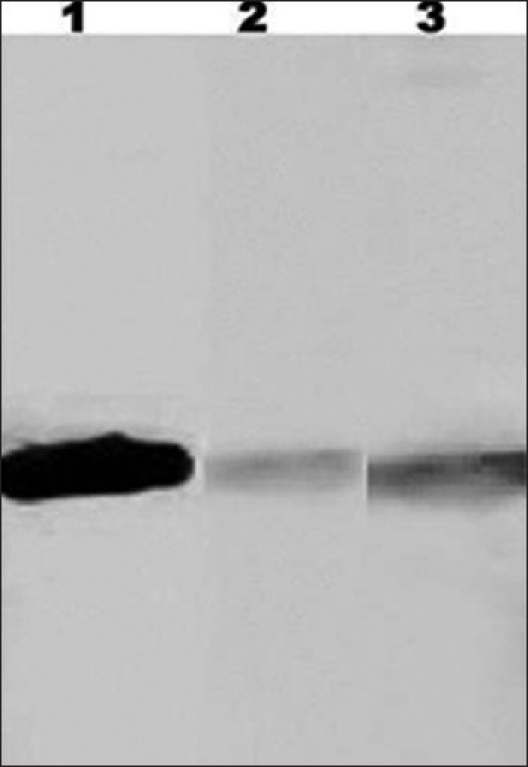
α -tubulin in human sperm detected by Western blot analysis. Lane 1 - Positive control i.e. purified bovine tubulin (Sigma chemicals) 5 mg/ml, 5 µl was loaded. Lanes 3 and 2 - α-tubulin in normozoospermic males and OA males, respectively. The protein band observed in OA cases is weak as compared to normozoospermic cases in lane 3

*α tubulin detection in OA men*: [Figure 2] shows that α tubulin is much lower in OA cases as compared to normozoospermic males.

**Figure 3:**
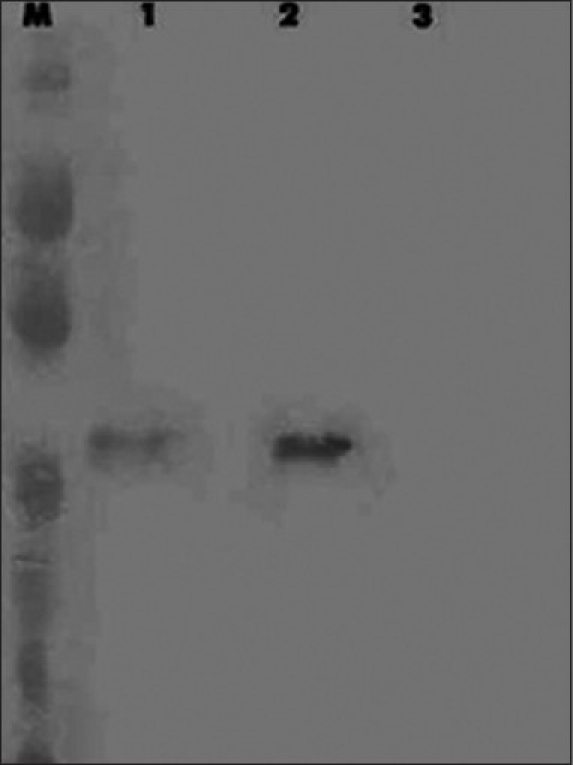
γ-tubulin in human sperm detected by Western blot analysis. Lane 2 and 1-γ-tubulin in normozoospermic and OA males, respectively. Similar to α tubulin the protein band is very weakly represented in OA cases. Lane 3 - Negative control (monoclonal antibody was substituted with 0.01 M PBS)

*γ tubulin detection in OA men:* γ-tubulin levels were significantly lower in OA cases as compared to normozoospermic males [Figure 3].

**Figure 4:**
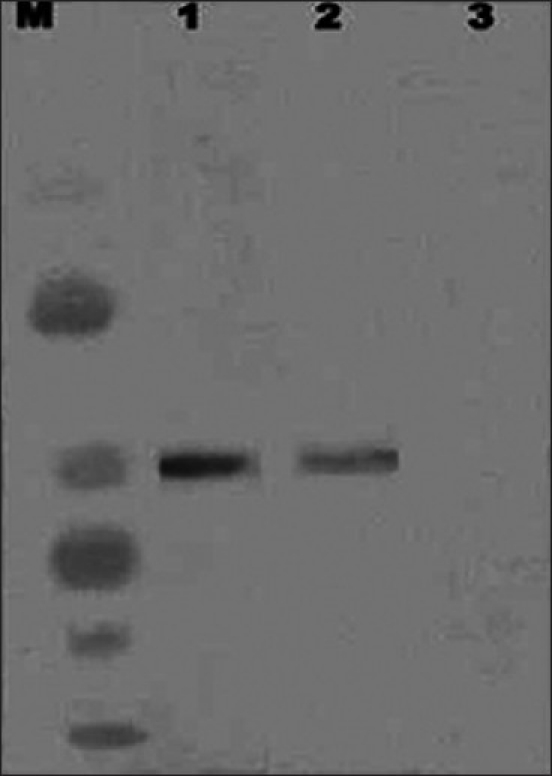
Centrin in human sperm detected by Western blot analysis. Lanes 1 and 2 - Centrins in normozoospermic and OA males. Lane M - Broad range rainbow protein molecular weight marker (12,000 – 2,25,000, Amersham Biosciences now GE Healthcare Lifesciences). Lane 3 - Negative control (monoclonal antibody was substituted with 0.01 M PBS)

*Centrin detection in OA men:* Similar to α and γ-tubulin, centrin levels were significantly lower in OA cases [Figure 4] as compared to normozoospermic males.

### Cell ELISA studies

Results of ELISA studies show significant decrease in α tubulin, γ-tubulin, and centrin [Figures 5a–c]. The levels of centrin were significantly lower (*P*<0.05), in OA men (Group 2) compared with normozoospermic (Group 1) men. Tubulin, both α and γ, were significantly lower in Group 2 (*P*<0.005) as compared with Group 1.

**Figure 5a:**
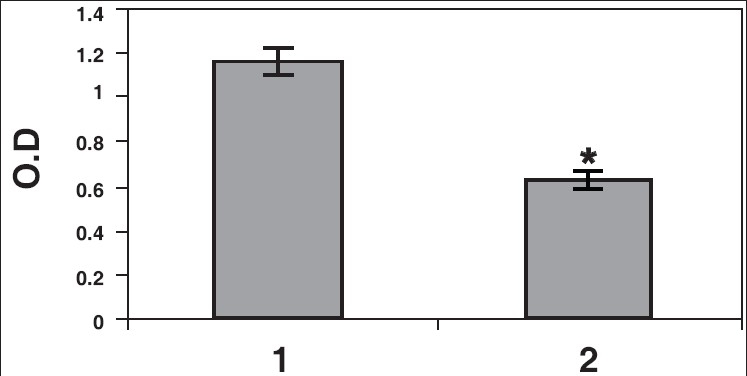
Sperm proteins detected by ELISA. Centrin levels were quantitated by ELISA in normozoospermic (1) and OA (2) males. The levels of centrin in OA males were significantly decreased (*P<0.05)

**Figure 5b:**
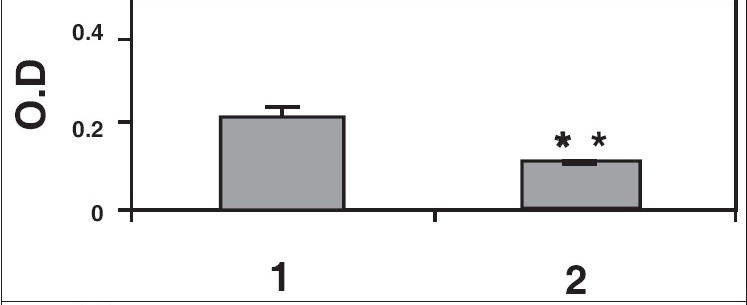
α-tubulin levels in normozoospermic (1) and OA (2) males. Reduction in α tubulin levels seen in OA males was statistically significant (**P<0.005) as compared to normozoospermic males

**Figure 5c:**
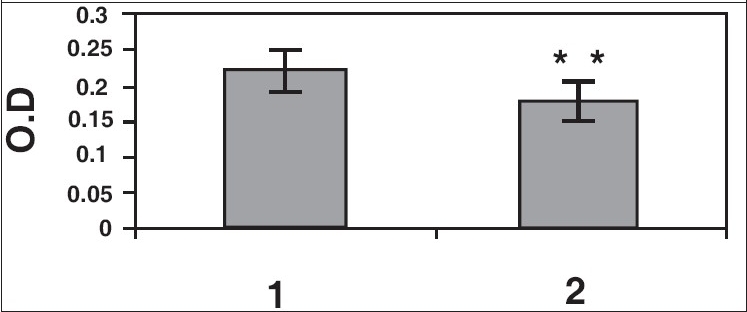
γ-tubulin levels in spermatozoa from normozoospermic (1) and OA (2) males. Reduction in γ tubulin in OA males was statistically significant (**P<0.005) as compared to normozoospermic males

## DISCUSSION

The centrosomes are the major microtubule organizing centers of cells. It is a truly central and main cellular station that directs, coordinates, and regulates most cellular functions either directly or indirectly through its microtubule organizing capabilities. In more recent years, staining of centrosomes with immunological probes confirmed that in most animal species except for the mouse dominant centrosomal material is contributed by sperm.[11,12] Direct assessment of sperm centrosome function is difficult, though scientists have used heterologous intracytoplasmic sperm injection (ICSI) system[13–16] to understand sperm centrosome function. These studies have indicated the importance of sperm centrosome function and correlate its dysfunction to infertility. However, precise information regarding the molecular and cellular events following the entry of sperm into the oocyte cytoplasm is limited.

Dysplasia of the fibrous sheath (DFS), a rare form of teratozoospermia, results in infertility. DFS sperm, which are immotile due to deformities from midpiece to tail[17,18] also, exhibit sperm centrosomal dysfunction; both of these abnormalities may cause infertility.[19,20] Failure of either fertilization or embryo development occurs in several patients, even after performing ICSI.[21] Low rates of sperm aster formation and oocyte activation were found in two other studies.[22,23]

Sperm centrosome dysfunction, zygote centrosome dysfunction, and polyspermy have been implicated in male infertility.[24] In humans, the functional proximal centriole is carried into the oocyte at the time of fertilization. Recent studies by Terada *et al*, 2007[25] have indicated the importance of sperm centrosomal function for the process of fertilization, the union of male and female genome inside the egg.

A recent study by Terada *et al*,[25] has assessed the oocyte activation ability and the centrosomal function of the patient’s sperm using bovine oocytes. They have found no difference in the oocyte activation ability between patient’s sperm and normal sperm. However, these researchers observed that sperm centrosomal function was low in the patient’s sperm compared with normal sperm, as assessed by the sperm aster formation rates in bovine oocytes. This suggested that their ability to process the fertilization after ICSI was lower, indicating the importance of sperm centrosomal function.

Since the centrosome is located in the sperm flagella, we were interested in determining whether there was any quantitative difference in the sperm centrosomal proteins in normozoospermic and oligoasthenozoospermic men. We have demonstrated lower levels of α and γ-tubulin by Western blot analysis in OA males as compared with normal men. The centrosomal protein γ-tubulin is vital in the growth of microtubules and is a part of a large complex, which is probably functional unit of MTOC that nucleates microtubules.[26,27] Biparental inheritance of γ-tubulin during human fertilization has been reported by Simerly *et al*.[28]

Oakley and Oakley[29] isolated the protein encoded by the microtubule interacting protein. This protein was neither α nor β tubulin but was γ-tubulin. The sequence similarities of these proteins are highly conserved. The well-studied γ-tubulin is a protein of the centrosome core structure.

γ-tubulin is a highly conserved protein in eukaryotes. It is vital for centrosome functions and elimination of γ-tubulin is lethal to cells and to the organisms[30] while elimination of components that binds to γ-tubulin has little effect on cell viability. Zheng *et al*,[31] reported that γ-tubulin is localized in the centrosome and may be a universal component of microtubule organizing center (MTOC) important for both nucleation and in defining polarity to the assembled centrosomal microtubules. The function of -tubulin was clearly determined by purification of the γ-tubulin ring complex (γ-TuRC) that revealed a ring shape and a substructure that were able to nucleate microtubule polymerization *in vitro*.[32] In some cases of teratospermia, human centrosomal function and the expression of pericentriolar protein was low. Expression of γ-tubulin in spermiogenesis might be related to centrosomal function during fertilization.[33] Remnants of γ-tubulin have been observed in human spermatozoa suggesting the role of paternal γ-tubulin in aster formation.[28] The sperm centrosome also has some γ-tubulin both around and within the centriole, represented by dense material.[34] Both paternal and largely maternal γ-tubulin evidently contributes to the zygote centrosome to restore its functionality.[35,36] Although the function of γ-tubulin in nucleating cytoplasmic and mitotic microtubules from organizing centers such as the centrosome and spindle pole body is well documented, its role in microtubule nucleation in the eukaryotic flagellum is unclear. Recent studies by Chan *et al*,[37] have demonstrated that γ-tubulin phosphorylation is very important for sperm motility.

The present study shows lower γ-tubulin expression in OA males as demonstrated by Western blot and cell ELISA suggesting that not only γ-tubulin is important for microtubule nucleation and cell polarity,[31] but also for aiding in motility of spermatozoa. With the previous studies[38] indicating a paternal inheritance, inheritance of defective centrosome may lead to abnormal cleavage and contribute to infertility. Poor centrosomal function of testicular spermatozoa has been shown to impair embryo development *in vitro* after intracytoplasmic sperm injection.[39]

We have for the first time demonstrated by ELISA a significantly lower level of centrin in sperm from OA cases as compared to normozoospermic males. This suggests the role of centrin in motility. Since centrin is a calcium binding protein[40] involved in microtubule severing during flagellar excision, it may be accrued that centrin plays a vital role during fertilization. Centrin might be important for the generation of the sperm flagellum, similar to the generation of axonemes of the motile cell organelles.[41] Zoran *et al*,[42] have proposed that an increase in calcium, transient, at fertilization, triggers the excision of the sperm axoneme from centrosome and increase in intracellular calcium,[43] this might be a centrin-induced uncoupling of the axoneme from basal body. Bornens *et al*,[44] have shown that increase in intracellular calcium also aids in the separation of centrioles at anaphase.

It is evident from the previous studies by Zoran *et al*,[42] that centrin has a role in the increase of intracellular calcium during the acrosome reaction. The absence or decrease of centrin content in the spermatozoa from OA cases may also hamper the acrosome reaction, which requires increased intracellular calcium levels, leading to failed fertilization. Centrin initiates calcium release in the oocyte during activation resulting in the excision of the sperm tail from the centrosome and transformation of basal body into a matured centrosome has been reported by Schatten.[35] Abnormal centrin expression has been shown in teratozoospermic males[45] where the spermatozoa exhibited abnormal alignment of the head-tail junction indicating severe centrosomal dysfunction. Sperm centrosome which introduces microtubule organization and promotes pronuclear opposition and first mitotic spindle formation may play a leading role in the post ICSI events in fertilization.

In conclusion, the present study demonstrates a lower centrosome protein expression in OA males as compared with normozoospermic men. Thus, the sperm centrosome from men of varying fertility status differs in their molecular composition. In depth studies of these proteins are warranted so as to design rational approaches for the diagnosis and treatment of male infertility.
